# Early growth response 1 (EGR1) is downregulated in peripheral blood from patients with major psychiatric disorders

**DOI:** 10.47626/2237-6089-2023-0749

**Published:** 2024-11-06

**Authors:** Giovana Bristot, Jacson Gabriel Feiten, Bianca Pfaffenseller, Gabriel Henrique Hizo, Gabriela Maria Pereira Possebon, Fernanda Endler Valiati, Jairo Vinícius Pinto, Marco Antonio Caldieraro, Marcelo Pio de Almeida Fleck, Clarissa Severino Gama, Márcia Kauer-Sant'Anna

**Affiliations:** 1 Hospital de Clínicas de Porto Alegre Centro de Pesquisa Experimental Laboratório de Psiquiatria Molecular Porto Alegre RS Brazil Laboratório de Psiquiatria Molecular, Centro de Pesquisa Experimental, Hospital de Clínicas de Porto Alegre, Porto Alegre, RS, Brazil.; 2 Universidade Federal do Rio Grande do Sul Porto Alegre RS Brazil Programa de Pós-Graduação em Bioquímica, Universidade Federal do Rio Grande do Sul (UFRGS), Porto Alegre, RS, Brazil.; 3 UFRGS Porto Alegre RS Brazil Programa de Pós-Graduação em Psiquiatria e Ciências do Comportamento, UFRGS, Porto Alegre, RS, Brazil.; 4 McMaster University Department of Psychiatry and Behavioral Neurosciences Hamilton ON Canada Department of Psychiatry and Behavioral Neurosciences, McMaster University, Hamilton, ON, Canada.; 5 Universidade Federal de Santa Catarina Hospital Universitário Florianópolis SC Brazil Hospital Universitário, Universidade Federal de Santa Catarina, Florianópolis, SC, Brazil.; 6 UFRGS Departamento de Psiquiatria Porto Alegre RS Brazil Departamento de Psiquiatria, UFRGS, Porto Alegre, RS, Brazil.

**Keywords:** Psychiatric disorders, bipolar disorder, schizophrenia, major depressive disorder, early growth response 1

## Abstract

**Objective:**

To evaluate relative expression of genes with the potential to translate environmental stimuli into long-term alterations in the brain, namely early growth response (EGR)1, EGR3, and cryptochrome circadian regulator (CRY)2 genes, in peripheral blood from patients with bipolar disorder (BD), schizophrenia (SZ), and major depressive disorder (MDD) and from healthy controls (HC).

**Methods:**

Thirty individuals ranging from 18 to 60 years old were recruited for each group (BD, SZ, MDD, or HC) from a Brazilian public hospital. These individuals’ peripheral blood was collected and EGR1, EGR3, and CRY2 gene expression analyzed by real-time polymerase chain reaction (qPCR).

**Results:**

EGR1 mRNA levels were significantly lower in psychiatric patients when compared to HC, but there were no differences for EGR3 or CRY2. Exploring the findings for each diagnosis separately, there were only significant differences between each of the diagnostic groups and controls for EGR1, which was lower in BD, MDD, and SZ compared to HC. No significant correlations were found between gene expression and clinical features.

**Conclusion:**

EGR1 is downregulated in psychiatric patients, regardless of diagnosis, and may be a potential common target in major psychiatric disorders. As a transcription factor, EGR1 modulates many other genes and participates in crucial neuronal and synaptic processes, such as plasticity, neurotransmitter metabolism, vesicular transport, and signaling pathways. The study of EGR1 and its upstream regulators might lead to potential new therapeutic targets in psychiatry.

## Introduction

Schizophrenia (SZ), bipolar disorder (BD), and major depressive disorder (MDD) are among the leading causes of disability worldwide and are usually associated with high morbidity and mortality.^1^ The pathophysiology underlying these complex disorders includes both genetic and environmental factors^2^ and the existence of a shared genetic predisposition between diagnoses has become increasingly evident, especially with regard to those reflecting similar clinical symptoms, risk factors, and drug therapies.^3^ As environmental factors have been considered important triggers for psychiatric illnesses, interest has grown in genes with the potential to translate environmental stimuli into long-term alterations in the brain, acting as key regulators of neuronal gene expression and neural plasticity. In this scenario, members of the immediate early gene (IEGs) transcription factors family, such as the early growth response (EGR)1 and the EGR3 genes, have been strongly suggested as potential mediators of the genetic and environmental influences in major psychiatric disorders.^4^

EGR1 and EGR3 play important roles in modulation of neuronal activity and neuroplasticity across the brain, participating in pathways related to learning and memory formation.^5–8^ They are expressed at basal levels throughout brain regions under normal physiological conditions and are rapidly induced by several stimuli. It is well known that EGR1 may be induced by cell stress, injury, neurotransmitters, cytokines, growth and differentiation factors, and other extracellular signals.^5^ EGR3 expression is also induced at high levels in response to environmental changes, such as stressful events and sleep deprivation.^9,10^

Acting as transcription factors, these genes modulate downstream targets, which play crucial roles for the normal functioning of the brain. This means that changes in EGR1 and EGR3 expression could potentially influence the activity of several biological pathways involved in the pathophysiology of psychiatric disorders. Indeed, EGR1 has been associated with mood disorders and with SZ,^4,11,12^ and has been identified as an important target in the response to treatment.^13,14^ In addition, a growing number of studies have suggested that EGR3 is potentially involved with relevant pathways associated with major psychiatric disorders.^15–20^

The day-night cycle represents another important stimulus when referring to environmental factors and its association with psychiatric illness is well described.^21^ The circadian system modulates various physiological activities such as metabolism, hormone secretion, cell proliferation, and apoptosis. Cryptochrome circadian regulator (CRY) 2 is one of these circadian clock genes involved in cell-cycle progression and DNA-damage checkpoint control.^22^ Psychiatric disorders are frequently associated with deregulation of circadian rhythm responses, such as cortisol secretion and sleep, and affective symptoms can be exacerbated by disruption of circadian rhythms.^23^ For example, one study reported that patients with BD have lower CRY2 expression when compared to controls^24^ and CRY2 has been associated with MDD and SZ^25–27^ and also with rapid cycling in BD.^28^

Considering the extensive and crucial physiological roles of these genes, dysfunction in their regulation could impair neuronal pathways and cognitive functions and may be reflected in poor outcomes seen in neuropsychiatric illness. Therefore, the aim of this study was to evaluate expression of EGR1, EGR3, and CRY2 genes in three severe mental illnesses using a transdiagnostic approach, to understand whether these genes show modified expression in major psychiatric disorders in comparison to controls, and whether they have diagnostic specificity for SZ, BD, or MDD.

## Methods

### Study design and participants

Four groups of subjects were studied: patients with BD, SZ, or MDD and healthy controls (HC). Each group consisted of 30 individuals with ages ranging from 18 to 60 years. Subjects were recruited from outpatient clinics at the Hospital de Clínicas de Porto Alegre (HCPA) and controls were recruited from the hemotherapy service blood bank at the same hospital, in Porto Alegre, state of Rio Grande do Sul, Brazil. Inclusion criteria for patients were diagnosis of a major psychiatric disorder (BD, SZ, or MDD), with no current substance use disorder, and absence of chronic inflammatory diseases or other severe medical conditions. Psychiatric disorder diagnoses were established by trained psychiatrists based on the criteria for BD and SZ set out in the Diagnostic and Statistical Manual of Mental Disorders, Fifth Edition (DSM-5), using the Structured Clinical Interview of the DSM-5, and based on DSM-IV criteria for MDD, using the Mini-International Neuropsychiatric Interview (MINI). Controls were individuals with no history of psychiatric or neurologic illnesses, no use of illicit substances, and absence of chronic inflammatory diseases or other severe medical conditions. One control group member did not answer the questionnaires properly and was excluded from the analyses for this reason.

All patients and controls were interviewed to assess sociodemographic variables related to sex, age, and education. Moreover, all patients underwent clinical interviews during which clinical variables were assessed, such as age of onset, duration of illness, hospitalizations, number of episodes (when applicable), suicide attempt, comorbidities, smoking, and alcohol and other substances use. Patients were also assessed using clinical scales as follows, for BD: (a) the Hamilton Depression Rating Scale (HDRS), (b) the Clinical Global Impression (CGI), (c) the Young Mania Rating Scale (YMRS), and (d) the MINI; for SZ: (a) the Calgary Depression Scale for Schizophrenia (CDSS), (b) the CGI, and (c) the Positive and Negative Syndrome Scale (PANSS); and for MDD: (a) the HDRS, (b) the CGI, (c) the MINI, (d) Beck's Depression Inventory I (BD), and (e) the Childhood Trauma Questionnaire (CTQ).

### Ethical considerations

The HCPA Research Ethics Committee approved the research protocol (2019-0025). All participants provided written informed consent prior to enrollment on the study.

### Total RNA isolation and complementary DNA (cDNA) preparation

Peripheral blood was collected from patients and controls in vacutainer tubes containing ethylenediamine tetraacetic acid (EDTA) and the whole blood was processed within 2 hours of collection. 500 μL of anticoagulated blood was added to 1.3 mL of RNA*later* stabilization solution and mixed thoroughly. Samples mixed with RNA*later* were stored at −80 °C until RNA isolation. RNA was extracted using a RiboPure-Blood Kit (Invitrogen – AM1928 – Carlsbad, USA) according to the manufacturer's protocol. RNA quantification was conducted using a Qubit RNA HS Assay Kit (Invitrogen – Q32855 – Carlsbad, USA) with the Qubit Fluorometer. Total RNA samples were spectrophotometrically scanned (at 260 and 280 nm) (Nanodrop ND-1000, Thermo Fisher Scientific, Wilmington, USA). The A260/A280 ratio was > 1.9, excluding relevant protein contamination. 300 ng of each RNA sample was used for cDNA synthesis using a High-Capacity cDNA Reverse Transcription Kit (Applied Biosystems – 4368814 – Carlsbad, USA) according to the manufacturer's instructions.

### Quantitative real-time polymerase chain reaction (qRT-PCR)

Five potential reference genes were tested – namely large ribosomal protein (RPLPO), 18S ribosomal RNA (RRN18S), beta-2-microglobulin (B2M), actin beta (ACTB), and glyceraldehyde-3-phosphate dehydrogenase (GAPDH) – to select the most stable gene (the one which exhibited the most stable expression levels in RNA isolated from peripheral blood) as the endogenous control. B2M performed best, so this housekeeping gene was run together with each target gene and used to perform normalization procedures in the analyses. Pre-designed TaqMan Gene Expression Assays (Applied Biosystems, Foster City, USA) were chosen for the target genes: EGR1 (Hs00152928_m1), EGR3 (Hs00231780_m1), and CRY2 (Hs00323654_m1). Approximately 70 ng of the cDNA was combined with Taqman PCR Master Mix (QuatroG, Porto Alegre, RS, Brazil – 100082), a pre-designed TaqMan Gene Expression Assay, and B2M. qRT-PCR was performed with a QuantStudio 3 Real-Time PCR System (Applied Biosystems - Singapore, Singapore) with a 96-well format for B2M and the three target genes. All PCR measurements were performed in triplicate. Control wells containing no cDNA template showed no amplification. The threshold cycle (Ct) was automatically determined from amplification plots and gene expression was quantified using the relative threshold method (Crt) with B2M as the endogenous control. Delta cycle relative threshold values (ΔCrt = Crt_target gene_ – Crt_endogenous control_) were calculated for each sample and 2^-ΔCrt^ values were included in the R software analysis (version 4.1.1).

### Statistical analyses

All data were analyzed using R (version 4.1.1) and the RStudio 1.4 interface. First, demographic and clinical variables were compared between the groups using the Kruskal-Wallis rank sum test, Pearson's chi-square test, and Fisher's exact test. Second, 2^-ΔCrt^ values were normalized using the bestNormalize R package (version 1.8.2) and the differences in normalized mRNA expression detected by qPCR were tested using multivariate analysis of variance (MANOVA) with post-hoc Tukey analysis, followed by analysis of variance (ANOVA) for each gene. Third, linear regression was used to confirm the differences among groups controlling for demographic variables (age, sex, and education). p < 0.05 was considered significant. Each gene was investigated to compare controls *versus* patients (regardless of their psychiatric diagnoses) and then for each psychiatric disorder separately *versus* controls and data were expressed as median ± interquartile interval. Correlations between mRNA levels and clinical parameters and psychiatric medications were tested with Pearson's correlation test. Receiver-operating characteristic (ROC) curves were plotted to evaluate the capacity of each gene to predict the groups.

## Results

### Demographic and clinical data

Demographic and clinical variables are presented in Table 1. There were significant differences in age, sex, and years of education between groups. Subsequent analyses were therefore controlled for these variables. There were no significant differences between the groups of patients in terms of duration of illness or CGI. Clinical assessments found that our sample showed moderate depressive symptoms in those with BD (HDRS = 17.83 [± 5.75]; YMRS = 2.47 [± 2.47]), moderate to severe depressive symptoms in those with MDD (HDRS = 21.30 [± 4.91]), and mild to moderate illness in those with SZ (PANSS = 61.04 [± 16.67]). All patients were taking psychiatric medications (antidepressants, mood stabilizers, antipsychotics, and/or benzodiazepines), and most were taking more than one psychiatric medication, as detailed in supplementary Figure S1. However, the small sample size in each medication group precluded study of medication effects on the outcomes. As expected for a transdiagnostic sample, medications were different in each diagnostic group, with some overlap (supplementary Table S1). In addition, it is noteworthy that 90% of patients with MDD were taking antidepressants (mostly selective serotonin reuptake inhibitors), 50% of individuals with BD were taking lithium, and 100% of patients with SZ were taking antipsychotics (83% of whom were taking clozapine).

**Table 1:** Demographic and clinical characteristics of participants

		Control (n = 29)	MDD (n = 30)	BD (n = 30)	SZ (n = 30)	p-value
Age, mean (SD)		39.69 (9.52)	47.14 (8.88)	43.73 (12.54)	42.10 (9.36)	0.038*
					
Sex, n (%)						< 0.001†
	Female	15 (52)	21 (70)	22 (73)	5 (17)	
	Male	14 (48)	9 (30)	8 (27)	25 (83)	
					
Years of education, mean (SD)		11.36 (3.96)	8.41 (4.74)	12.77 (4.16)	9.21 (3.19)	< 0.001*
Age at onset in years, mean (SD)		n/a	28.97 (13.00)	21.30 (9.22)	21.10 (6.33)	0.032*
Duration of illness in years, mean (SD)		n/a	18.00 (12.69)	22.43 (12.22)	21.41 (9.20)	0.300*
CGI, median (interquartile range)		n/a	5 (4.0-5.0)	5 (4.0-5.0)	4 (3.5-5.0)	0.117*

BD = bipolar disorder; CGI = Clinical Global Impression Scale; MDD = major depressive disorder; n/a = not applicable; SD = standard deviation; SZ = schizophrenia. Columns show mean (SD) for all categories, except for sex and CGI. Data are presented as n (%) for sex, and median (median of lower half – median of upper half) for CGI.

* Kruskal-Wallis rank sum test;

† Pearson's chi-square test.

### EGR1, EGR3, and CRY2 gene expression in psychiatric patients versus HC

**Figure 1:**
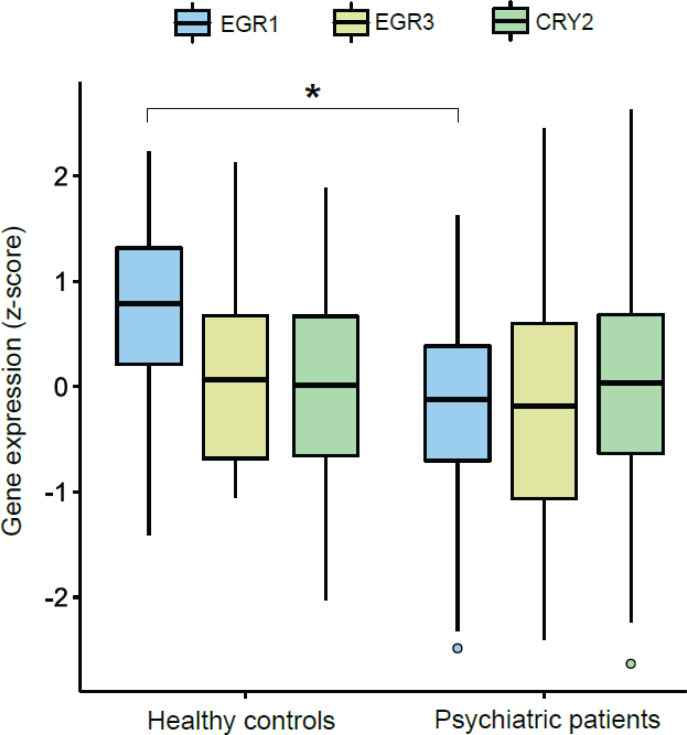
EGR1, EGR3 and CRY2 mRNA levels (z-score) in psychiatric patients (n = 90) and healthy controls (n = 29). Bars represent the median ± interquartile interval. CRY2 = cryptochrome circadian regulator 2; EGR1 = early growth response 1; EGR3 = early growth response 3. * p < 0.001 according to multivariate analysis of variance using Wilks’ statistic.

Using RT-qPCR, we compared EGR1, EGR3, and CRY2 mRNA levels in whole blood between psychiatric patients and controls. For our first analysis, from a transdiagnostic perspective, we compared EGR1, EGR3, and CRY2 mRNA levels between all psychiatric patients regardless of diagnostic group and controls. Figure 1 and supplementary Table S2 show that EGR1 mRNA levels were significantly lower in psychiatric patients compared to HC (Bonferroni p-value < 0.001), but there was no difference for EGR3 (Bonferroni p = 0.738) or CRY2 (Bonferroni p = 1.00) transcripts when comparing these two groups. Linear regression results showed that there were no gender-related differences in EGR1 mRNA levels among the participants (p = 0.918).

### EGR1, EGR3, and CRY2 gene expression for each diagnosis (BD, MDD, and SZ) versus HC

**Figure 2:**
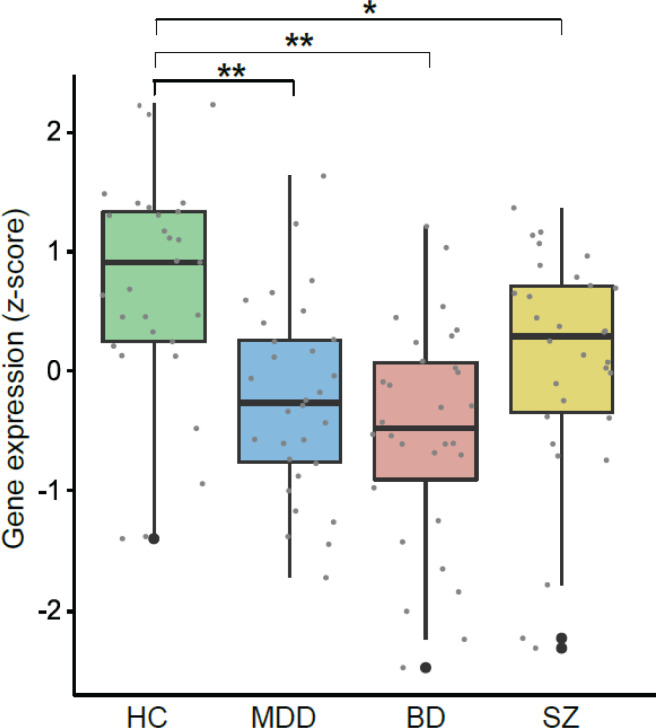
EGR1 mRNA levels (z-score) in HC (n = 29), MDD (n = 30), BD (n = 30) and SZ (n = 30) groups. Bars represent the median ± interquartile interval. EGR = early growth response 1; BD = bipolar disorder; HC = healthy controls; MDD = major depressive disorder; SZ = schizophrenia. ** p < 0.001 and * p = 0.026 according to multivariate analysis of variance followed by Tukey's post-hoc test.

Further, we examined whether changes in EGR1 mRNA levels or in EGR3 or CRY2 expression were particularly associated with any of the three diagnoses. When we grouped the psychiatric patients by diagnosis (BD, MDD, or SZ), there was a significant difference between groups only for EGR1 (p < 0.001) (Table 2). EGR1 transcripts were significantly lower in BD (p < 0.001), MDD (p < 0.001), and SZ (p = 0.026) compared to HC controlling for age, sex, and education (Figure 2 and supplementary Table S3).

**Table 2:** MANOVA results of genes assessed as a function of four groups (HC, MDD, BD, and SZ)

Gene	F value	Num df	Den df	p-value	Bonferroni p-value
EGR1	9.74	3	116	< 0.001	< 0.001
EGR3	0.93	3	116	0.429	1.000
CRY2	0.491	3	116	0.689	1.000

BD = bipolar disorder; CRY2 = cryptochrome circadian regulator 2; Den df = denominator degrees of freedom; EGR1 = early growth response 1; EGR3 = early growth response 3; HC = healthy controls; MANOVA = multivariate analysis of variance; MDD = major depressive disorder; Num df = numerator degrees of freedom; SZ = schizophrenia.

### EGR1, EGR3, and CRY2 gene expression to differentiate patients from HC

**Figure 3:**
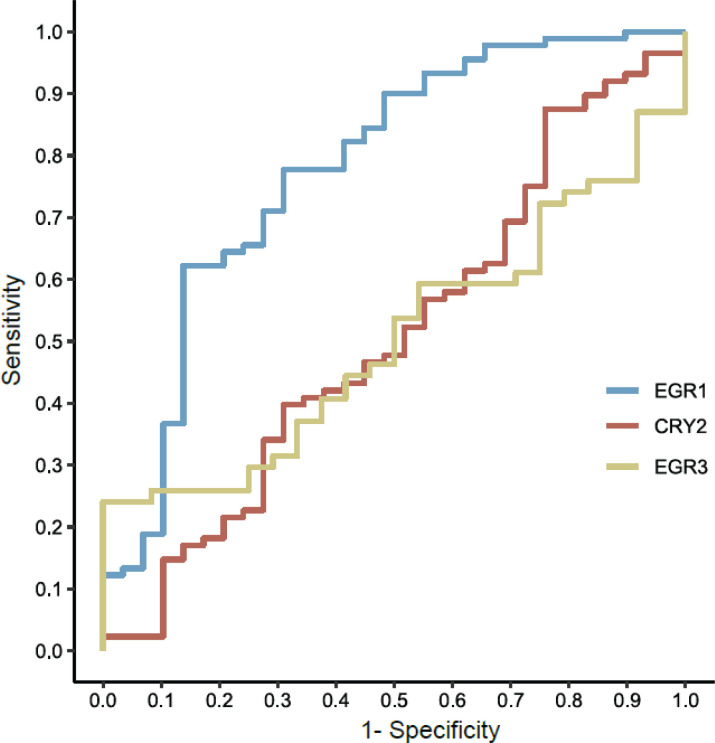
ROC curve analysis for EGR1, CRY2 and EGR3 between psychiatric patients and controls. ROC analysis showed an AUC of 0.78 for EGR1, 0.50 for EGR3, and 0.49 for CRY2. AUC = area under the curve; CRY2 = cryptochrome circadian regulator 2; EGR1 = early growth response 1; EGR3 = early growth response 3; ROC = receiver operating characteristic.

In line with the idea of a transdiagnostic approach, we also tested the ability of each of the three genes to differentiate patients from controls, regardless of the psychiatric diagnosis (BD, MDD, or SZ). The results showed classifications that achieved an area under the curve (AUC) of 0.78 (0.67-0.88), with 0.91 sensitivity and 0.45 specificity for EGR1, AUC of 0.50 (0.37-0.63), with 0.46 sensitivity and 0.54 specificity for EGR3, and AUC of 0.49 (0.37-0.62), with 0.5 sensitivity and 0.48 specificity for CRY2. None of these genes achieved good scores, but nonetheless once again EGR1 showed the best performance for differentiating psychiatric patients from HC (Figure 3).

**Figure 4:**
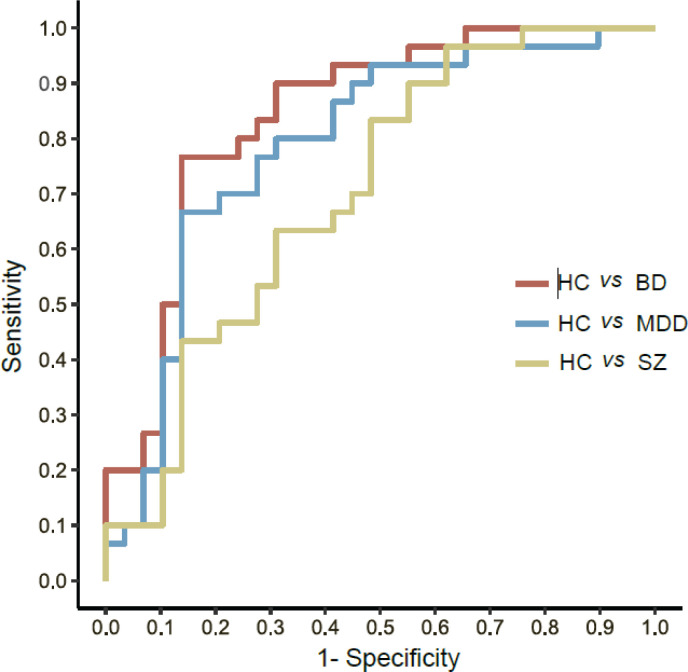
ROC curve analysis for EGR1 between controls and each disorder individually. ROC analysis showed an AUC of 0.84 for BD vs. controls; 0.79 for MDD vs. controls; and 0.70 for SZ vs. controls. AUC = area under the curve; BD = bipolar disorder; EGR1 = early growth response 1; HC = healthy controls; MDD = major depressive disorder; ROC = receiver operating characteristic; SZ = schizophrenia.

Moreover, as EGR1 showed the best performance, we decided to explore further each diagnosis separately *versus* HC to check if AUC would improve. We observed that a better AUC score was only obtained for HC vs. BD, with an AUC of 0.84 with 0.70 sensitivity and 0.86 specificity. HC vs. MDD achieved an AUC of 0.79 with 0.63 sensitivity and 0.86 specificity, and HC vs. SZ achieved an AUC of 0.70 with 0.36 sensitivity and 0.86 specificity (Figure 4). We also attempted to use EGR1 data to differentiate between the three disorders (BD, MDD, and SZ), but this approach did not return good results (supplementary Figure S2).

### Effects of medications and clinical characteristics on EGR1, EGR3, and CRY2 mRNA levels

No significant correlations were found between ΔCrt values for the three genes analyzed and clinical parameters, such as specific scales for each disorder. Regarding BD, for example, HDRS, CGI, YMRS, and MINI had no correlation with ΔCrt values for EGR1, EGR3, or CRY2. No significant correlations were observed in SZ between CDSS, CGI, or PANSS and the ΔCrt values of these three genes, nor in MDD between HDRS, CGI, MINI, BDI, or CTQ and the ΔCrt values for EGR1, EGR3, or CRY2. Furthermore, logistic regression also failed to show any significant associations between ΔCrt values and duration of illness, age at onset, years of education, or use of any psychiatric medication.

## Discussion

Researchers searching for biological markers of psychopathology have used different approaches, from investigating a common pathway in a transdiagnostic approach to identifying molecular signatures of major diagnosis, such as SZ, BD, and MDD. Investigation of biomarkers in psychiatry is highly relevant since they represent biological features of the diseases that can be objectively assessed and might be useful in clinical practice to support both diagnosis and prediction of clinical outcomes related to the course of the illness. Biomarkers could also potentially suggest which therapies are most likely to be effective for a particular patient, facilitating personalized treatment.^29^ In this study, EGR1, EGR3, and CRY2 were selected based on their potential to mediate gene-environment effects on psychopathology and based on previously reported association with psychiatric disorders,^5,11,12,24,28,30–33^ including studies from our group.^15,20^

Our major finding is the downregulation of EGR1 expression in major psychiatric disorders compared to HC when applying a transdiagnostic approach, indicating that EGR1 may be part of a putative pathway underlying a common physiopathology of such illnesses. When considering the disorders individually, the decrease in EGR1 expression was consistent in BD, MDD, and SZ. EGR1 is a transcription factor involved in neuron maturation^34^ via N-methyl-D-aspartate (NMDA)-dependent hippocampal synaptic plasticity,^35^ essential for memory consolidation and reconsolidation.^36^ EGR1 is expressed throughout the brain, maintaining baseline expression levels in areas connected to the control of social and emotion-driven behavior, sensitivity to reward, long-term memory, and cognition.^31^ EGR1 transcription is indirectly regulated by stress or learning tasks, leading to changes in neuronal activity, secretion of hormones, and release of growth factors.^31^ Furthermore, EGR1 has the potential to regulate an array of target genes implicated in biological functions linked to synaptic plasticity, such as metabolism of neurotransmitters, vesicular transport, and signaling pathways.^31,37^

Therefore, impairments in the functioning of EGR1 may be critical to the cognitive symptoms often described in psychiatric conditions, especially considering that EGR1 acts in the integration of environmental signals at the synaptic plasticity level to modulate central processes such as learning and memory.^31^ Sun et al.^38^ recently predicted that EGR1 plays a crucial role in epigenetic brain programming, which refers to the process of how life events can leave sustainable marks in the brain.

Our findings showing reduced EGR1 mRNA levels in the blood of SZ individuals compared to controls corroborate other studies.^39,40^ However, a study investigating transcriptional signatures in major psychiatry disorders reported EGR1 upregulation in fibroblasts and whole blood from SZ patients experiencing elevated psychotic states compared to controls and this altered EGR1 expression was not observed in MDD or BD.^41^ Although findings conflict, this evidence suggests involvement of the EGR1 gene in the pathophysiology of SZ.

In addition, it has been shown that EGR1 is significantly downregulated in brain regions of SZ patients, especially the prefrontal cortex.^12,42,43^ In MDD, similar data were reported by Covington et al.,^44^ who found reduced EGR1 expression in the medial prefrontal cortex in non-medicated subjects, as well as in depressed patients refractory to treatment, consistent with a deficit in neuronal activity in this brain region reported in MDD. In preclinical studies, EGR1 mRNA and protein levels were reported to be reduced in the frontal cortex of mice submitted to social isolation stress,^45^ and chronic stress caused reduced expression of EGR1 in the hippocampus^46^ and medial prefrontal cortex,^44^ besides an increase of EGR1 mRNA in the lateral amygdala of rodents.^47^ Interestingly, a recent study highlighted downregulation of EGR1 as the top dysregulated gene among thousands of genes involved in depression in a combined portrait of depression, suggesting impaired neuronal activity.^48^ Furthermore, in a recent study using a systems biology approach, we found that EGR1 was consistently repressed in the dorsolateral prefrontal cortex of BD, MDD, and SZ patients.^15^

EGR1 plays an important role in synaptic plasticity and may participate in the augmentation of apical and total dendritic spine density, together with activity-regulated cytoskeleton-associated protein (*Arc)*, which is another IEG and is considered a synaptic plasticity gene.^49^ Studies have indicated that synaptic plasticity is reduced in BD, MDD, and SZ, as evidenced by loss of dendritic spines and dendrites or decreased synapse numbers.^50–52^ In this sense, considering that EGR1 controls several other genes and, ultimately, entire biological pathways, reduced expression may contribute to the impaired synaptic plasticity reported in psychiatric disorders.

It is well known that SZ, BD, and MDD have a relevant genetic component and that environmental factors also play crucial roles in the development and pathophysiology of these illnesses.^31^ Changes in the expression of EGR1 can vary across the central nervous system depending on the intensity, duration, and nature of the stress.^5^ Therefore, EGR1 may represent a connection between early environmental stimuli (such as stressful experiences) and predisposition to psychiatric disorders in adolescence and adulthood, acting through pathways involving neuronal response to stress via dendritic spine arrangement. We know that adverse life events are associated with mood episodes and age of psychopathology onset, as well as with severity of symptoms and comorbidities.

Differently from EGR1, we did not find any difference between controls and psychiatric patients in EGR3 expression levels. The EGR family includes four members and the differences in the gene expression pattern of EGR3 and EGR1 in patients and HC in our study could be related to their distinct upstream regulatory pathways, activators, and targets.^31^ We also did not find any significant changes in CRY2 expression between groups. It is possible that medications could have influenced their differential expression between groups. It is essential to point out that our sample is drawn from a tertiary care hospital, a referral center for treatment of severe psychiatric illness on the public health care system, thus most patients are at an advanced stage of illness progression and are taking multiple drugs, which can interact, impacting gene expression through numerous biological pathways, and this may have influenced the results. For instance, EGR3 can be upregulated by antipsychotic medications^53^ and CRY2 by lithium.^54^ Potential medication effects on the mRNA levels of the three genes studied cannot be ruled out. Regarding the effects of medication on EGR1, we believe that its gene expression levels could have been observed to be even more reduced in a drug-free scenario, since medications potentially increase EGR1 mRNA levels. For instance, studies have shown that EGR1 transcripts were upregulated by lithium in mouse frontal cortex^13^ and by valproic acid in neural stem cells,^55^ which indicates this pathway is modulated by these drugs, considering that EGR1 stimulates synaptic plasticity and these mood stabilizers reverse synaptic plasticity deficits.^56–58^ Moreover, it has been suggested that antipsychotics induce EGR1 expression. Increased levels of EGR1 expression were observed in the striatum and nucleus accumbens of rodents after acute administration of clozapine or haloperidol,^59^ or after acute administration of olanzapine, asenapine, or haloperidol,^60^ as well as with acute and chronic low dose lurasidone.^61^

Our study has some limitations that merit comment. First, we measured EGR1, EGR3, and CRY2 expression in whole blood, and it is not possible to correlate this with the expression pattern in the brain. Second, it was not possible to control or evaluate the influence on gene expression levels of medications and licit drugs (including alcohol) taken by the patients. Third, our patient sample is composed of individuals from a tertiary care hospital, which means they are usually critically ill patients and frequently have comorbidities. Fourth, as a cross-sectional study, we cannot suggest if the molecular changes in EGR1 observed were present before illness onset or if these changes are a consequence of the chronic course of the psychiatric disorders. Fifth, the small sample size may restrict some analyses. Therefore, studies are warranted with larger sample sizes, aiming to replicate these results and shed light on the influence of these genes and related pathways on major psychiatric disorders.

Finally, the results from our study together with the current literature suggest that the EGR1 pathway has a key role in mediating the environmental influences and the gene expression regulation associated with major psychiatric disorders. EGR1 and its pathway could be a potential novel target for therapeutic interventions and deserves further investigation. It is of note that EGR1 expression is regulated by complex mechanisms, and future research in the field of psychiatry could include investigation of its direct or indirect regulators.
