# Supplementary material for: Early growth response 1 (EGR1) is downregulated in peripheral blood from patients with major psychiatric disorders
Source: Trends Psychiatry Psychother. 2024 Nov 6;46:e20230749. doi: 10.47626/2237-6089-2023-0749 (PMC11815351; doi:10.47626/2237-6089-2023-0749)
Supplement: Supplementary file 1 [file 2238-0019-trends-46-e20230749-suppl01.pdf]

http://doi.org/10.47626/2237-6089-2023-0749

Figure S1 - Psychiatric medications taken by the full sample (n= 90), (detailed individually for each disorder – MDD (n= 30), BD (n= 30) and SZ (n= 30)). Each column refers to a patient, lines represent different psychiatric medications, and the numbers between parentheses indicate the number of patients under the use of such psychotropic drug.

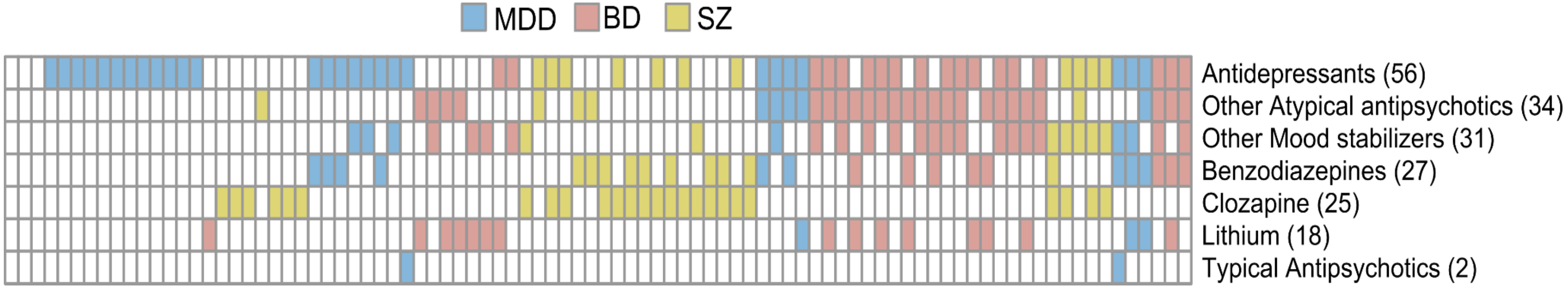

BD = bipolar disorder; MDD = major depressive disorder; SZ = schizophrenia.

http://doi.org/10.47626/2237-6089-2023-0749

Table S1 - Psychotropic drugs taken by patients.

	MDD (n = 30)	BD n = 30	SZ n = 30	p-value
Antidepressants, n (%)[a]	27 (90%)	18 (60%)	11 (37%)	< 0.001
Lithium, n (%)[a]	3 (10%)	15 (50%)	0 (0%)	< 0.001
Valproic acid, n (%)[a]	3 (10%)	13 (43%)	7 (23%)	0.012
Anticonvulsants, n (%)[a]	6 (20%)	5 (17%)	7 (23%)	0.8
Benzodiazepine, n (%)[a]	9 (30%)	8 (27%)	10 (33%)	0.9
Antipsychotics (total), n (%)[a]	7 (23%)	24 (80%)	30 (100%)	< 0.001
Clozapine, n (%)[a]	0 (0%)	0 (0%)	25 (83%)	< 0.001
Other atypical antipsychotics, n (%)[a]	5 (17%)	24 (80%)	5 (17%)	< 0.001
Typical antipsychotics, n (%)[b]	2 (7%)	0 (0%)	0 (0%)	0.326

BD = bipolar disorder; MDD = major depressive disorder; SZ = schizophrenia.

[a] Pearson's chi-squared test.

[b] Fisher's exact test.

Table S2 - Multivariate analysis results of mRNA levels in control group and psychiatric patients' group.

	F value	Num DF	Den DF	p-value	Bonferroni p-value
EGR1	22.464	1	118	< 0.001	< 0.001
EGR3	1.36	1	118	0.246	0.738
CRY2	0.02	1	118	0.887	1.000

Num DF = numerator degrees of freedom; Den DF = denominator degrees of freedom; EGR1 = early growth response 1; EGR3 = early growth response 3; CRY2 = cryptochrome circadian regulator 2.

Table S3 - Multiple pairwise comparisons of the multivariate analysis results of EGR1 assessed as a function of four groups (healthy control, MDD, BD, and SZ).

Comparison	Estimate	Conf. low	Conf. high	p-value adj.
Control - MDD	-0.925	-1.532	-0.318	< 0.001
Control - BD	-1.193	-1.800	-0.587	< 0.001
Control - SZ	-0.606	-1.212	0.001	0.026
MDD - BD	-0.268	-0.875	0.339	0.658
MDD - SZ	0.320	-0.287	0.927	0.518
BD - SZ	0.588	-0.019	1.195	0.061

BD = bipolar disorder; Conf. = confidence; EGR1 = early growth response 1; MDD = major depressive disorder; SZ = schizophrenia.

http://doi.org/10.47626/2237-6089-2023-0749

Figure S2 - ROC curve analysis for EGR1 between the disorders (BD vs. MDD; BD vs. SZ; MDD vs. SZ). ROC analysis showed an AUC of 0.57 for BD vs. MDD, with 0.63 sensitivity and 0.30 specificity; 0.7 for BD vs. SZ, with 0.37 sensitivity and 0.30 specificity; and 0.64 for MDD vs. SZ, with 0.37 sensitivity and 0.37 specificity.

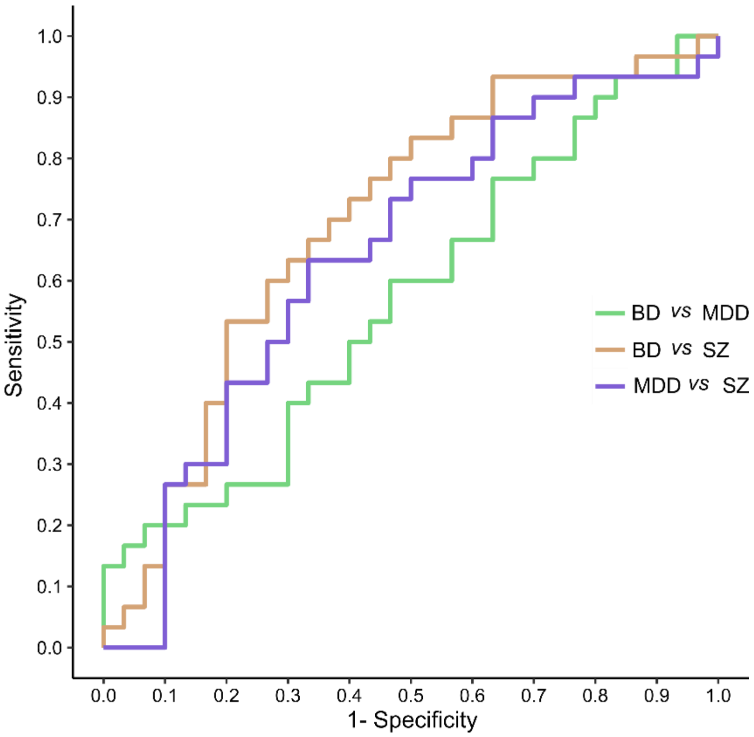

AUC = area under the curve; BD = bipolar disorder; EGR1 = early growth response 1; HC = healthy controls; MDD = major depressive disorder; ROC = receiver operating characteristic; SZ = schizophrenia.
